# In-vitro analysis of stress dynamics in polyamide and metal acrylic distal extension removable partial dentures

**DOI:** 10.1186/s12903-025-06969-1

**Published:** 2025-10-09

**Authors:** Asmaa Ejaz Khan, Asif Ali Shah, Farasat Iqbal, Gotam Das, Asif Ali, Waled Abdulmalek Alanesi

**Affiliations:** 1 Department of Prosthodontics, de Montmorency College of Dentistry, Lahore, Pakistan; 2 Department of Prosthodontics, Rashid Latif Dental College, Lahore, Pakistan; 3 Interdisciplinary Research Centre in Biomedical Materials (IRCBM), COMSATS University Islamabad, Lahore Campus, Islamabad, Pakistan; 4 Department of Prosthodontics, College of Dentistry, King Khalid University, Abha, Saudi Arabia; 5 Research Unit Plasma Technology (RUPT), Department of Applied Physics, Ghent University, Ghent, Belgium; 6 Department of Operative Dentistry, Faculty of Dentistry, University of Science and Technology, Inmaa city, Aden, Yemen

**Keywords:** Bilateral distal extension, Removable partial denture, Stress distribution, Polyamide

## Abstract

**Background:**

This study aimed to determine and compare the stresses transmitted by metal acrylic and polyamide removable partial dentures (RPDs) on free-end saddle areas.

**Methods:**

Twenty metal acrylic and polyamide removable partial dentures were made. The stresses transmitted on the free end saddle area were determined and compared by using the strain gauge resistance method, in which sensors were installed in the epoxy resin cast. The load was applied on removable partial dentures with the underlying cast through the universal testing machine. Data was collected through the connected strain meter and computer. The analysis was done using ANSYS version 15, and the results were analyzed.

**Results:**

The polyamide distal extension removable partial dentures transmit higher but even stresses on the free end saddle area compared to metal acrylic distal extension RPDs. The forces transmitted by polyamide distal extension base RPDs distribute an even load on the ridge, whereas metal acrylic distal extension base RPDs distribute an uneven load on the ridge. P value equal to and < 0.05 was considered significant, and our results showed insignificant statistical differences.

**Conclusion:**

Stress distribution in polyamide distal extension removable partial dentures is even compared to metal acrylic distal extension removable partial dentures. Even force distribution is less damaging to the bone and surrounding tissues.

**Supplementary Information:**

The online version contains supplementary material available at 10.1186/s12903-025-06969-1.

## Introduction

Removable prosthesis is indicated to restore facial aesthetics and masticatory function, which in return improves the quality of life of edentulous patients. Treatment options for replacing missing teeth are removable partial dentures, fixed partial dentures, or dental implants [1, 2]. Distal extension base removable partial dentures (RPDs) is a typical treatment for patients who have lost their posterior teeth. RPDs with distal extension bases (DEB) are associated with problems related to differential support. These RPDs with DEB can cause harm in direct and indirect ways [3]. Distal extension RPDs exhibit a composite type of support gained from teeth, mucosa, and residual alveolar ridge. This differentiating support of partial dentures is associated with problems of support and stability and is challenging for providing a comfortable, satisfactory prosthesis [2, 4].

With the emergence of acrylic polymers and cobalt-chromium alloys, RPDs became popular years ago. Polymethyl methacrylate (PMMA) has been used as a denture base material since the 1930s. These tissue-supported acrylic RPDs have created a revolution in the provision of RPDs [5]. Tooth Tissue Supported metal acrylic RPDs provide support by occlusal rest. However, these dentures are subjected to forces acting laterally, obliquely, and apically (along three axis), causing torque action on the abutment tooth and ridge resorption [3, 5].

The critical component in the restoration of distal extension saddle areas by metal acrylic RPDs is the material’s rigidity which causes leverage. The use of a flexible material can solve this problem because the load of force is shifted from the denture’s design elements to the base material. A flexible RPD lowers leverage effects while maintaining the denture’s support and retention [6]. Distal extension RPDs perform complex stress dynamics since the support of DEB requires not only hard tissue but also soft tissue. A framework is taken in this paper to examine stress distribution in distal extension RPDs where polyamide and metal-acrylic materials were integrated [2]. This draws inspiration from nature’s streamlined designs to provide insight on how to achieve functional symbiosis between materials and biological tissues. The elastic nature of polyamide resembles the natural elasticity of oral structures, while the rigidity and durability of metal-acrylic frameworks are well-known. This research examines how applying principles through this material comparison can improve stress management, reduce tissue overload, and improve clinical outcomes, thus paving the way for better-designed prosthetic modalities [7].

A distal extension prosthesis made of polyamide denture base material may be helpful in eliminating problems associated with metal framework distal extension base RPDs, such as excessive stresses on the abutment teeth and aggravation of residual ridge resorption [4, 7]. The flexibility of base material has the functional benefit of shifting load from the prosthesis design elements to the base material. Without compromising support or retention, a flexible partial denture lowers the leverage effects of its extensions because the stress distribution is automatically balanced and eliminates the requirement for an occlusal rest [7, 8]. The function of occlusal rest is to compensate for damaging stresses that are produced by the fulcrum effect of rigid connectors. As flexible denture base material absorbs the masticatory forces by compressing within itself, fewer forces are transferred onto the supporting structures, thus maintaining and preserving their integrity. The material’s elasticity also helps to equalize masticatory stresses across the entire ridge rather than just at particular support points [9, 10].

The strain gauge resistance method is a useful tool for evaluating stress distribution in different types of prosthesis. This has been already used for the evaluation of stress analysis study of different prosthetic options using a single posterior implant for the management of mandibular distal extension saddle and stress analysis study of two treatment modalities rehabilitating distal extension cases with few remaining natural teeth [11–14]. This strain gauge resistance method can also be used for in-vitro comparative evaluation of stress distribution in polyamide and metal acrylic distal extension removable partial dentures. There has not been any study regarding the comparison of stress distribution in polyamide and metal acrylic distal extension removable partial dentures. The aim of the study is to evaluate and compare the stress distribution patterns transmitted to the free-end saddle areas by polyamide and metal acrylic distal extension removable partial dentures using strain gauge analysis. The hypothesis of study is no significant difference in the stress distribution transmitted by polyamide and metal acrylic distal extension removable partial dentures to the free-end saddle areas.

## Method

The removable partial dentures, metal acrylic andpolyamide is made in Prosthodontics department de’ Montmorency College of Dentistry incollaboration with Dar lab Lahore. The laboratory testing is done in IRCBM department Comsats,The removable partial dentures, metal acrylic andpolyamide is made in Prosthodontics department de’ Montmorency College of Dentistry incollaboration with Dar lab Lahore. The laboratory testing is done in IRCBM department Comsats,

### Fabrication of cast

**Fig. 1:**
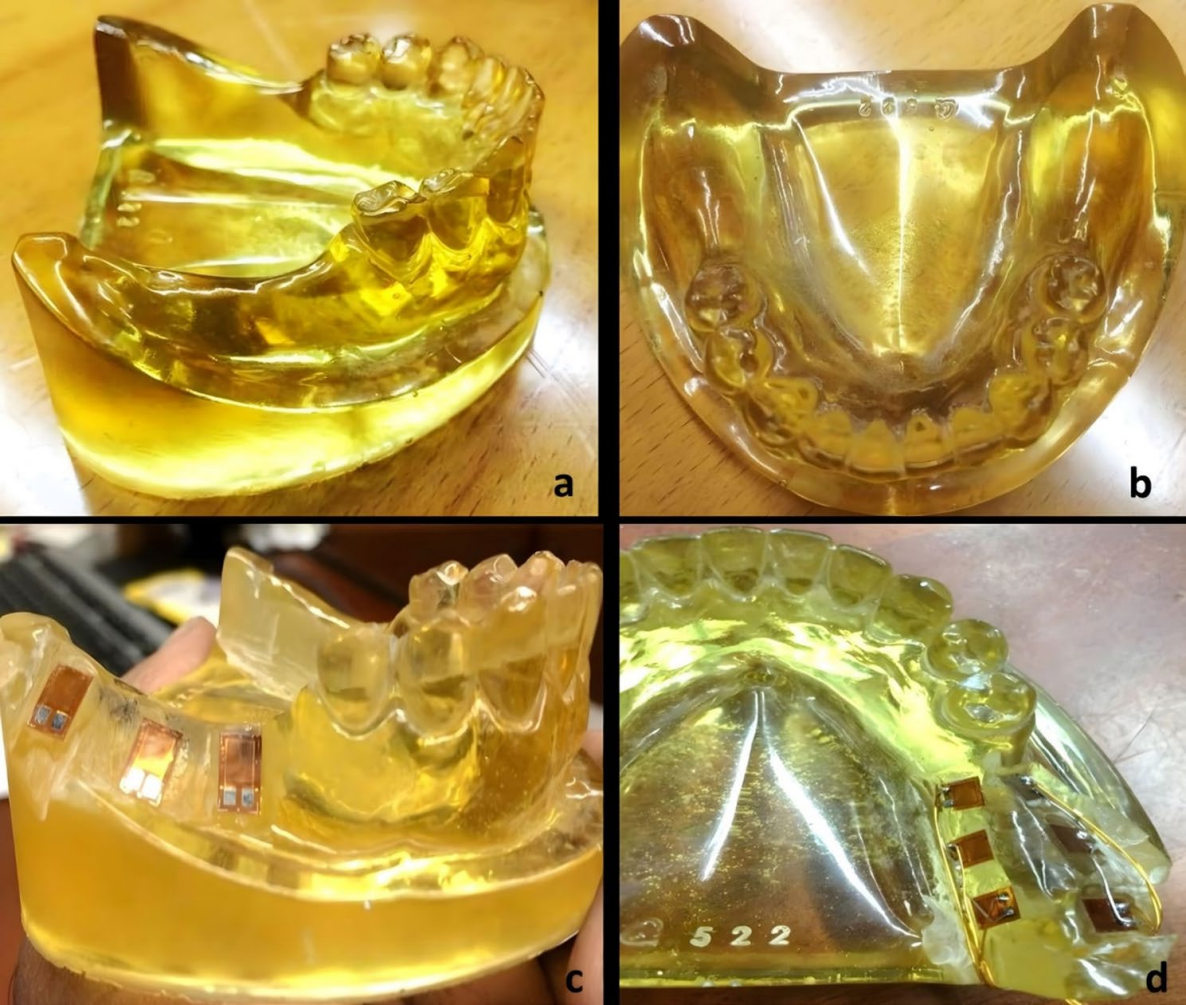
Epoxy Resin cast, Epoxy resin cast (lateral view), strain gauges installed in epoxy resin cast and Strain gauges and connection

A commercially available rubber mold of mandibular bilateral free-end distal extension base with teeth presents up to the second premolar was used. This mold was poured with epoxy resin material to fabricate single the required cast [15] as shown in Fig. 1. The cast is removed from the mold and is properly finished (Fig. 1b). Small channels were prepared in the epoxy resin cast at different areas to receive the strain gauges (Fig. 1c). The length and width of the channel were 2 × 2 mm. For the cementation of six strain gauges a strain gauge adhesive was used. Three strain gauges were placed on the buccal and three on the lingual sides of the alveolar ridge. A total of twelve strain gauges were placed on both ridges. This cast with strain gauges installed in it was then connected through a common pin to an electric circuit. (Fig. 1d).

### Fabrication of metal acrylic removable partial dentures

**Fig. 2:**
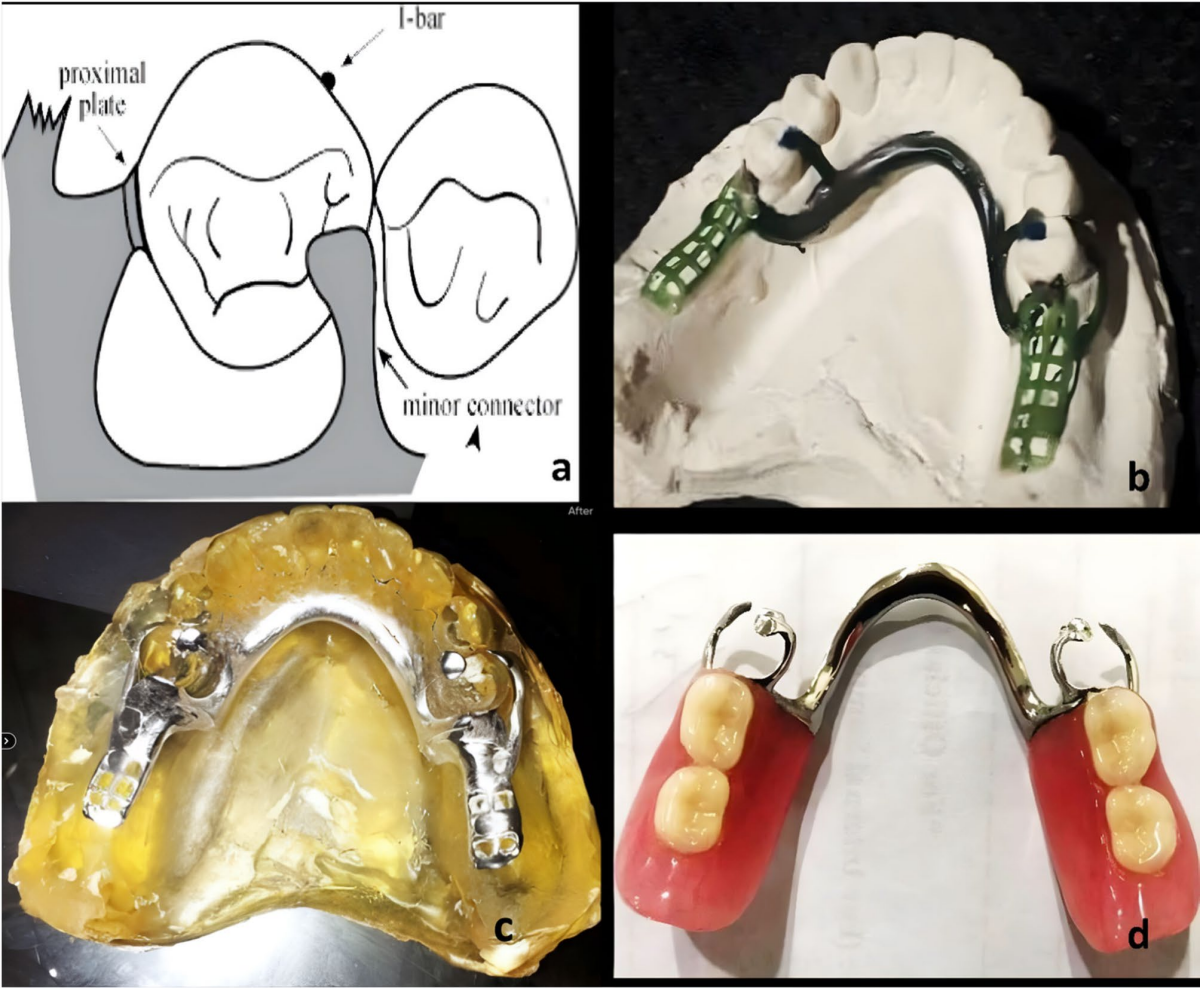
Design of RPD in a, waxing up of framework in b, Casted Co-Cr framework properly seated on epoxy resin cast c, Finished and Polished metal acrylic RPD d

For design 1, a traditional Co-Cr metal framework (SUPER 6 Argen, USA) was made using a standard protocol with dentures based on the edentulous space made of PMMA (Lucitone 199 - DENTSPLY). RPI design (Mesial rest, proximal plate, and I-bar) with long guide planes and lingual plate acting as a major connector Fig. 2a.

### Fabrication procedure

Transferring the design and modeling with preformed wax patterns was done (Fig. 2a and b). Lay mold on its side in the furnace, which is cold or preheated at 240 C. Pre-heat crucible simultaneously (1000–1080 C) according to wax pattern and manufacture instructions of investment material. Release centrifuge when the last ingot collapses into the melt and there is a uniform surface.

### Fabrication of polyamide removable partial denture

Polyamide removable partial denture with major connector, denture base, buccal clasps engaging the buccal undercut of second premolars, all made up of valplast with no occlusal rest (Fig. 10).

### Fabrication procedure

Drawing the design on the secondary cast and transferring this approved version to the master cast was achieved. ‘T’ shape holes were made (diatorics) in teeth of selected shade. The holes were made for the mechanical retention of acrylic teeth (Dental trading corp) to a flexible denture base. The holes may be made before arranging teeth or removing the teeth from the mould after dewaxing. If wax is not completely removed from holes, flexible material may not flow properly into holes from the cartridge, thereby affecting the retention of teeth with denture base. This technique of retention of acrylic teeth with a Polyamide denture base is known as the Retento-Grip tissue bearing technique.

Dewaxing was done by placing flasks in hot water for 3 to 5 min to soften the wax after investing in a particular flask developed for the injection-molding process. The flask was opened and flushed with clean boiling water to remove all the wax residue. The flask margin was checked and ensured that both flask halves fit together with intimate metal contact. A thin coat of separating agent was applied to the model and allowed the model to dry completely.

**Fig. 3:**
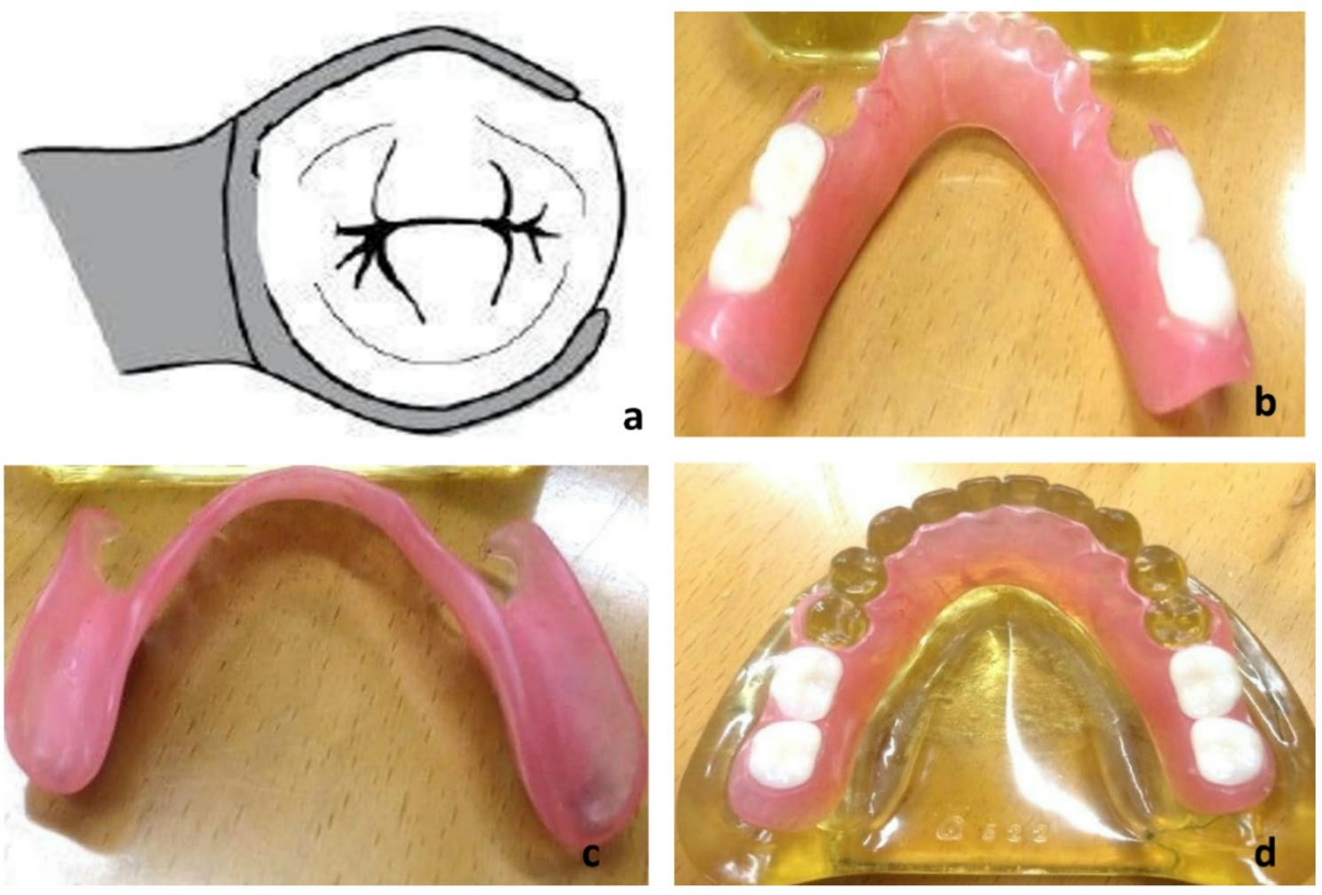
Design of Polyamide RPD (Buccal clasp engaging buccal undercut of second premolar with no occlusal rest a, Valplast RPD (Polished and tissue surfaces) b & c, Valplast RPD finished and polished, properly seated on epoxy resin cast

A suitable-sized cartridge was chosen and sprayed with silicone spray then it is placed in a cartridge carrier which was then placed in an electric cartridge furnace. It was used for softening of polyamide denture base material. The application of spray prevents the adhesion of the cartridge with the cartridge carrier and allows smooth separation. The material should be plasticized for 15 to 20 min at 550 to 560 °F (Valplast, Vertex Holland, Netherlands) (the softening temperature is different for different types of flexible denture base material). Maintain this temperature for 15 to 20 min. The time between removing the cartridge assembly from the furnace and injection should be less than 1 min. If it takes longer, the cartridge will begin to cool and may result in partial or no injection. The pressure should be maintained for 3 to 5 min. The material flows through the sprue into the mold. At this point, the flask was opened, and the prosthesis was retrieved. The flasks should not be opened immediately to prevent distortion of the prosthesis. As we have used a successful injection system, the injection pressure should be 100 psi, and the injection time should be 1 min. The finishing of the prosthesis was done after retrieving it. Finally, high luster shine was achieved by polishing the cake. The polishing cake was used with a dry buff to develop a highly bright/luster surface. Figure 3b shows the polished surface of polyamide mandibular RPD. Figure 3c shows the tissue surface of polyamide RPD as in Fig. 3d.

### Procedure for evaluation of stress

**Fig. 4:**
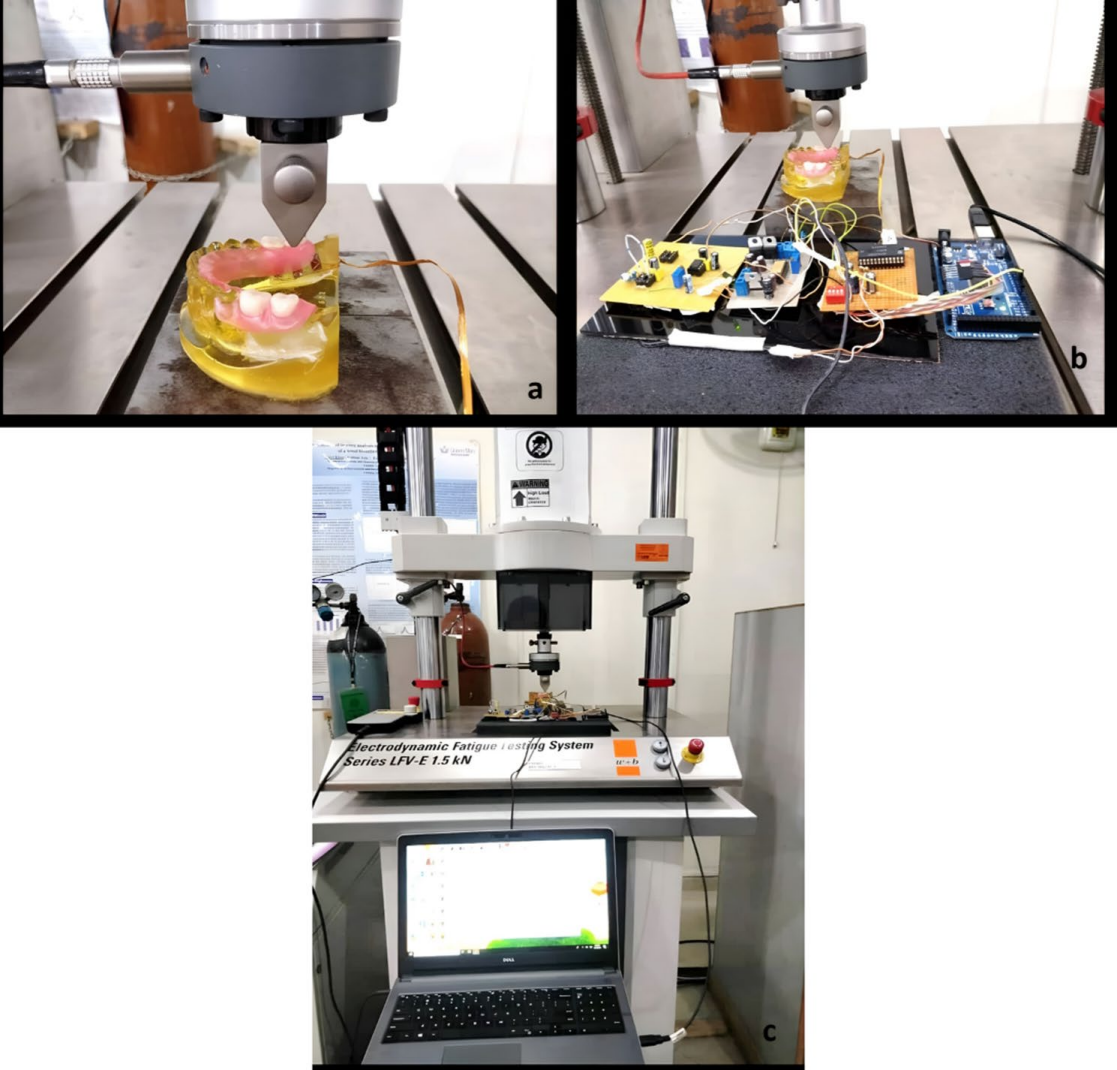
RPD properly seated on epoxy cast placed on measuring table of Universal Testing Machine a, Cast connected to the strain measuring device b, Whole assembly (cast attached to strain meter which is attached to computer and placed under Universal testing machine**)**

Both types of metal acrylic and polyamide RPDs were seated on the duplicated epoxy resin casts those having pre-installed strain sensors. Seating and positioning of removable partial dentures were ensured. One by one, each design was placed on the measuring table/machine base (Fig. 4a). The model was attached to the base of the universal testing machine (Norwood, MA, USA) in a horizontal plane and is connected to a computer through computer-aided software (Fig. 4b and c).

### Load application

An ascending load of 0–250 N was applied with the loading jig device on the occlusal surface of the 1 st and 2nd molar. The device measured the strain on every Newton and then gave the data to the software pre-installed in the computer. A period of rest of about 5–6 min was given between each loading for heat dissipation. In Fig. 4, the distal extension removable partial denture was seated on an epoxy resin cast, and this cast was placed on the measuring table of the universal testing machine. The triangular-shaped stainless-steel jig was used to apply the load. The RPD was seated on an epoxy resin cast and placed on the measuring table of the universal testing machine, which was further connected to the self-designed strain measuring device. The whole assembly was being shown in which load was being applied on the distal extension removable partial denture, connected to the strain measuring device which was attached to the laptop on which strain values were recorded every 1 N.

.

## Results

*For* *Sensor 1*, the comparison of stress transmission between metal acrylic and polyamide removable partial dentures (RPDs) revealed notable variations across the different sensors. At lower force levels (19 N to 115 N), there were no statistically significant differences in stress transmission between the two denture materials, with p-values well above 0.05. Specifically, the polyamide RPDs exhibited minimal or slightly increased stress transmission compared to metal acrylic RPDs; for example, at 19 N, the polyamide group showed a mean stress of −0.17 ± 5.91 N, while the metal acrylic group showed 4.54 ± 3.14 N, with a p-value of 0.29.

As the applied force increased, the polyamide RPDs began to transmit higher stress values compared to metal acrylic RPDs. This trend became more pronounced from 154 N onward. At 183 N, the polyamide RPDs transmitted 19.57 ± 6.12 N versus 11.9 ± 4.78 N for the metal acrylic RPDs (*p* = 0.162). While this was not statistically significant, the difference in transmitted force increased further at higher force levels.

Statistically significant differences emerged at 231 N and 250 N. At 231 N, the polyamide RPDs transmitted a mean stress of 26.99 ± 2.9 N compared to 16.34 ± 3.09 N in the metal acrylic group, with a p-value of 0.012. Similarly, at 250 N, the polyamide RPDs showed a mean stress of 29.88 ± 3.16 N versus 14.94 ± 4.74 N for the metal acrylic RPDs, with a highly significant p-value of 0.010. These results indicate that polyamide RPDs transmit significantly greater stress to the supporting tissues than metal acrylic RPDs at higher occlusal loads, (Table 1; Fig. 1).

**Table 1:** Comparison of the transmitted stress (Average strain values in percent %) between groups at sensors 1,2, and 3

Force	Sensor 1			Sensor 2			Sensor 3		
	Metal acrylic RPD	Polyamide RPD	*P*-Value	Metal acrylic RPD	Polyamide RPD	*P*-Value	Metal acrylic RPD	Polyamide RPD	*P*-Value
19	4.54 ± 3.14	−0.17 ± 5.91	0.29	−0.7 ± 5.65	1.91 ± 6.41	0.62	3.03 ± 8.17	3.05 ± 7.42	0.99
38	4.06 ± 2.90	0.56 ± 7.97	0.51	0.67 ± 8.69	2.2 ± 9.08	0.84	2.84 ± 7.76	3.66 ± 12.81	0.92
57	3.95 ± 4.20	2.12 ± 9.73	0.78	2.22 ± 6.71	−0.4 ± 7.99	0.68	3.31 ± 7.07	3.13 ± 12.11	0.98
77	5.06 ± 3.32	3.35 ± 7.44	0.73	2.38 ± 5.81	6.41 ± 7.71	0.51	5.37 ± 5.14	7.27 ± 9.91	0.78
96	5.3 ± 4.16	7.78 ± 9.54	0.70	2.12 ± 7.94	9.04 ± 10.69	0.42	5.39 ± 9.16	11.75 ± 11.29	0.49
115	4.57 ± 9.43	9.69 ± 9.77	0.55	4.56 ± 11.46	9.22 ± 11.53	0.64	6.2 ± 11.96	12.52 ± 13.82	0.58
124	9.8 ± 7.13	13.48 ± 6.36	0.54	9.43 ± 10.27	13.65 ± 8.01	0.60	11.6 ± 9.25	17.64 ± 11.24	0.51
154	11.78 ± 6.69	16.57 ± 4.85	0.37	11.39 ± 12.39	17.49 ± 5.97	0.48	13.07 ± 8.38	18.39 ± 9.08	0.49
183	11.9 ± 4.78	19.57 ± 6.12	0.16	15.01 ± 6.55	20.24 ± 6.78	0.39	15.45 ± 5.48	22.89 ± 7.60	0.24
192	14.88 ± 3.22	22.42 ± 4.88	0.08	17.69 ± 2.38	21.69 ± 4.93	0.27	18.35 ± 3.11	24 ± 7.89	0.31
212	13.82 ± 6.10	24.13 ± 3.32	0.06	18.46 ± 3.92	24.66 ± 5.64	0.19	18.55 ± 5.59	25.09 ± 5.22	0.21
231	16.34 ± 3.09	26.99 ± 2.90	0.01*	19.58 ± 5.29	28.54 ± 6.32	0.13	19.2 ± 4.86	28.15 ± 6.79	0.13
250	14.94 ± 4.74	29.88 ± 3.16	0.01*	18.72 ± 7.39	29.37 ± 6.30	0.13	17.17 ± 8.41	29.85 ± 5.51	0.09

*Sensor 2* recorded strain values under progressively increasing force levels (from 19 N to 250 N) for both Metal Acrylic Removable Partial Dentures (RPDs) and Polyamide RPDs. The mean and standard deviation (SD) of strain values were compared, and paired sample t-tests were conducted to evaluate statistical differences at each force level.

At lower forces (19–77 N), the strain values in both RPD types fluctuated with no consistent trend. For example, at 19 N, Metal RPD showed a slight negative strain (− 0.7 ± 5.65), while Polyamide RPD showed a small positive strain (1.91 ± 6.41), with no significant difference (*p* = 0.625). Similar non-significant differences were observed at 38 N, 57 N, and 77 N (p-values ranging from 0.51 to 0.843).

**Fig. 5:**
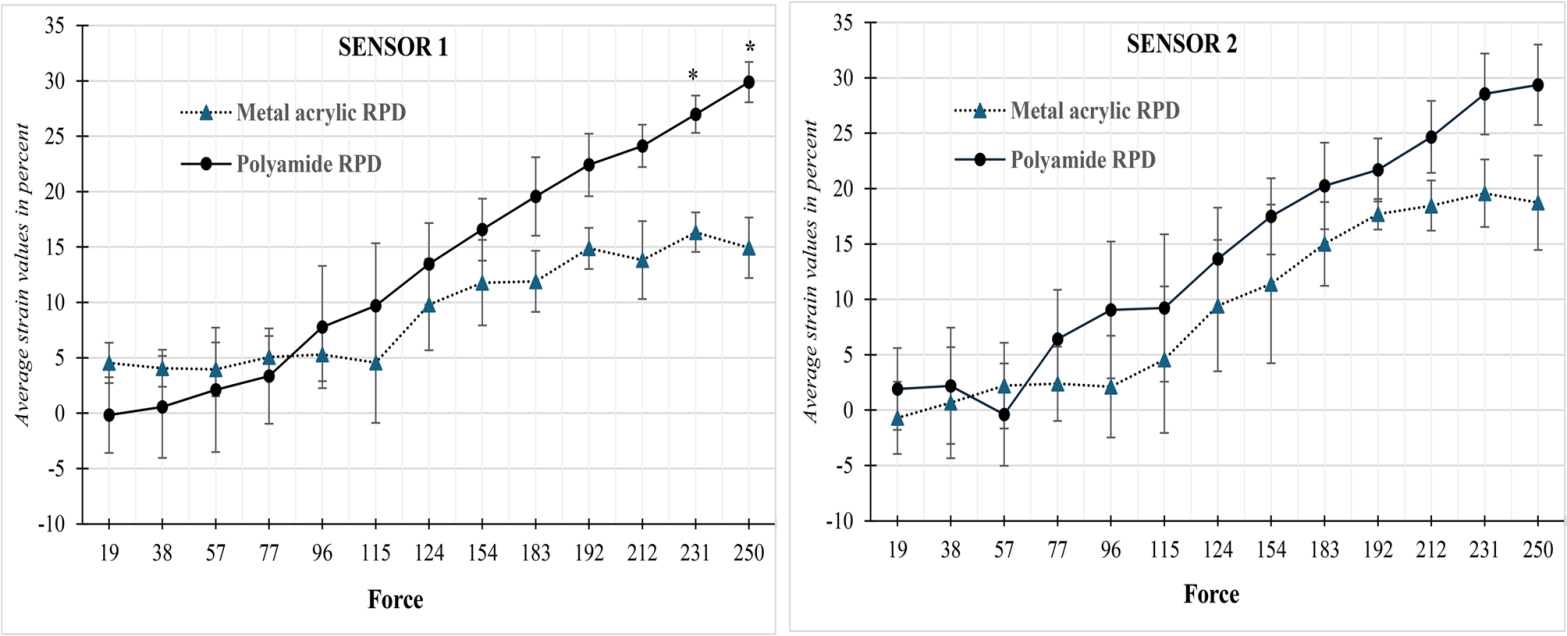
Comparison of transmitted strain between Metal acrylic and Polyamide RPD at sensors 1 and 2

From 96 N to 154 N, both denture types showed increasing strain values. Polyamide RPDs consistently showed higher mean strain compared to Metal Acrylic RPDs, though none of these differences were statistically significant. For instance, at 124 N, Polyamide RPD recorded 13.65 ± 8.01, higher than Metal RPD’s 9.43 ± 10.27, but *p* = 0.604, indicating no significant difference. At high force levels (183 N to 250 N), the gap in strain values between Polyamide and Metal RPDs widened. At 250 N, Metal RPD recorded 18.72 ± 7.39, while Polyamide RPD recorded 29.37 ± 6.30. Despite this numerical difference, the p-value was 0.130, still not reaching statistical significance. (Table 1; Fig. 5 )

**Fig. 6:**
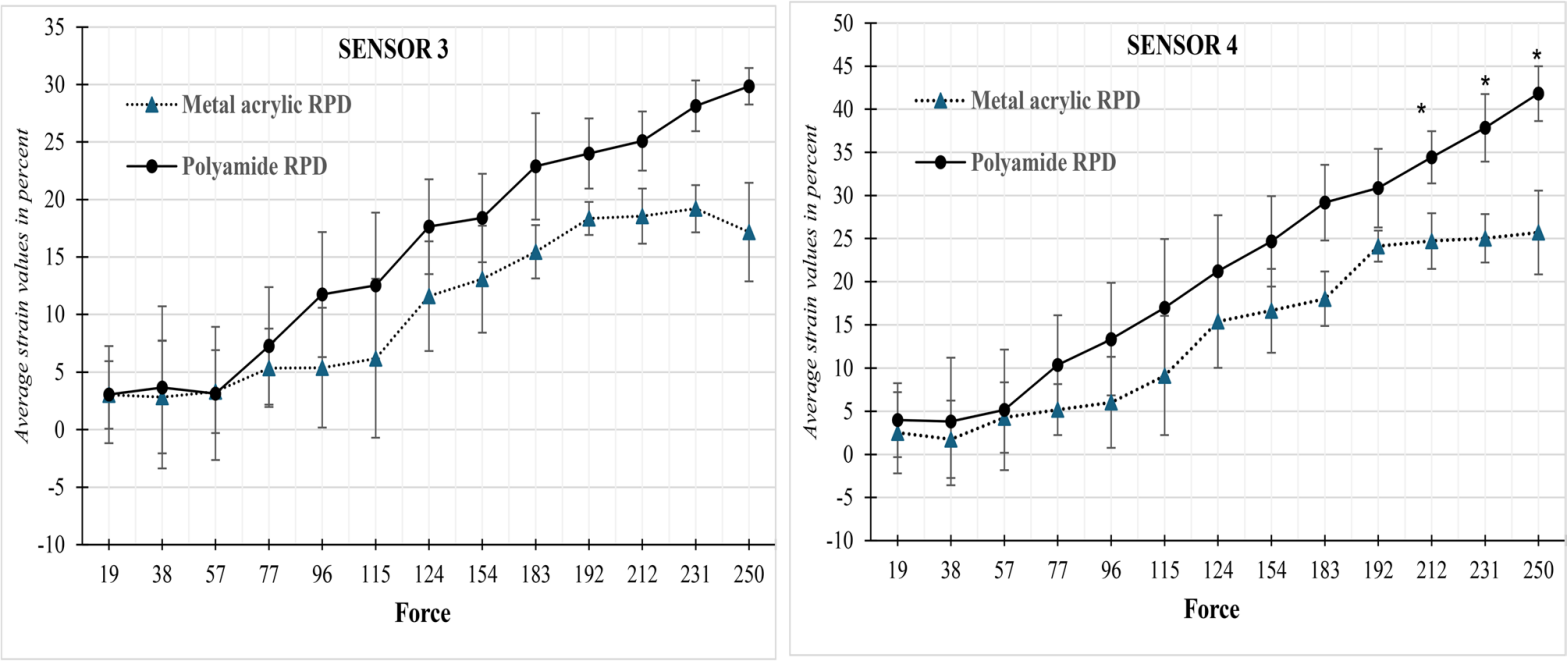
Comparison of transmitted strain between Metal acrylic and Polyamide RPD at sensors 3 and 4

*Sensor 3* measured the strain transmitted by metal acrylic and polyamide removable partial dentures (RPDs) under various applied forces, ranging from 19 N to 250 N. The data shows that the low forces (19 N to 77 N), both denture types transmitted similar levels of stress, with negligible differences in mean strain values and very high p-values (e.g., *p* = 0.998 at 19 N and *p* = 0.783 at 77 N), indicating no statistically significant difference. As force increased (from 96 N onward), polyamide RPDs began to transmit higher mean strain compared to metal acrylic RPDs. For instance, at 124 N, the polyamide RPD transmitted 17.64 ± 11.24 µstrain versus 11.60 ± 9.25 µstrain for the metal acrylic RPD; at 250 N, the strain was 29.85 ± 5.51 µstrain for polyamide versus 17.17 ± 8.41 µstrain for metal acrylic as shown in Table 1; Fig. 6

Despite the increasing trend in force transmission with polyamide RPDs at higher forces, none of the comparisons reached statistical significance (all p-values > 0.05). However, the p-values decreased as the force increased (e.g., *p* = 0.094 at 250 N), suggesting a potential emerging trend that might reach significance in a larger sample.

*Sensor 4* recorded the strain transmitted by metal acrylic and polyamide removable partial dentures (RPDs) at increasing force levels ranging from 19 N to 250 N. The findings indicate a progressive trend:

At Lower Forces (19–96 N): Both RPD types showed low to moderate strain transmission, with no significant differences. The mean strain values for polyamide RPDs were slightly higher than metal acrylic RPDs, but p-values remained well above 0.05 (e.g., *p* = 0.79 at 19 N, *p* = 0.386 at 96 N), indicating no statistically significant difference.

At Moderate Forces (115–192 N): The polyamide RPDs began to transmit noticeably more strain compared to metal acrylic RPDs. For example, at 154 N, the polyamide RPD transmitted 24.67 ± 6.65 µstrain, compared to 16.64 ± 8.07 µstrain with the metal acrylic RPD. Still, the p-values in this range (e.g., *p* = 0.255 to *p* = 0.116) suggest that the differences were not statistically significant yet but showed a strengthening trend. At Higher Forces (212–250 N): Statistically significant differences emerged:

At 212 N, polyamide RPD transmitted 34.43 ± 4.45 µstrain versus 24.72 ± 4.14 µstrain in metal acrylic (*p* = 0.050). At 231 N, the difference widened (polyamide: 37.85 ± 3.82, metal acrylic: 25.02 ± 3.57, *p* = 0.013). At 250 N, strain transmission was highest, with polyamide RPD reaching 41.83 ± 2.75 µstrain, significantly more than 25.71 ± 7.44 for metal acrylic RPD (*p* = 0.024) as shown in Table 2; Fig. 6

**Table 2:** Comparison of the transmitted stress (Average strain values in percent %) between groups at sensors 4, 5 and 6

Force	Sensor 4			Sensor 5			Sensor 6		
	Metal acrylic RPD	Polyamide RPD	* P*-Value	Metal acrylic RPD	Polyamide RPD	* P*-Value	Metal acrylic RPD	Polyamide RPD	*P*-Value
19	2.51 ± 5.09	3.98 ± 7.33	0.79	2.38 ± 4.36	3.08 ± 7.07	0.891	2.6 ± 4.62	1.51 ± 8.08	0.848
38	1.75 ± 8.48	3.81 ± 12.19	0.822	5.26 ± 8.00	5.09 ± 9.23	0.982	1.67 ± 7.00	−0.19 ± 11.24	0.82
57	4.27 ± 6.26	5.16 ± 10.01	0.903	3.87 ± 5.10	8.15 ± 8.71	0.503	−2.17 ± 5.92	−0.54 ± 10.28	0.823
77	5.2 ± 5.89	10.38 ± 8.82	0.445	6.65 ± 2.23	15.87 ± 8.33	0.18	0.06 ± 6.65	3.41 ± 10.29	0.66
96	6.04 ± 9.0	13.35 ± 9.4	0.386	6.51 ± 10.48	18.44 ± 12.20	0.268	1.57 ± 10.36	2.69 ± 11.15	0.653
115	9.13 ± 11.96	16.98 ± 10.96	0.449	10.19 ± 9.06	19.16 ± 11.04	0.338	0.24 ± 10.46	3.61 ± 8.85	0.652
124	15.38 ± 8.25	21.2 ± 7.14	0.408	13.98 ± 7.79	24.15 ± 8.76	0.211	2.3 ± 8.9	5.72 ± 6.18	0.613
154	16.64 ± 8.07	24.67 ± 6.65	0.255	14.46 ± 8.38	25.9 ± 10.4	0.212	0.97 ± 9.16	4.45 ± 7.48	0.637
183	18.01 ± 4.0	29.18 ± 8.02	0.097	16.93 ± 3.10	31.53 ± 8.82	0.054	1.47 ± 4.26	6.52 ± 4.75	0.243
192	24.11 ± 2.51	30.86 ± 5.28	0.116	18.79 ± 5.93	33.85 ± 9.25	0.076	−0.16 ± 6.82	7.18 ± 5.81	0.229
212	24.72 ± 4.14	34.43 ± 4.45	0.050*	20.93 ± 4.86	38.03 ± 10.64	0.064	1.97 ± 4.39	8.6 ± 4.00	0.125
231	25.02 ± 3.57	37.85 ± 3.82	0.013*	23.15 ± 5.60	42.14 ± 8.60	0.033*	0.2 ± 4.38	10.49 ± 3.29	0.031*
250	25.71 ± 7.44	41.83 ± 2.75	0.024*	22.19 ± 7.71	48.77 ± 5.47	0.008*	−2.25 ± 8.47	12.54 ± 2.65	0.045*

*In Sensor 5*, the strain values measured for both metal acrylic and polyamide removable partial dentures (RPDs) show an increasing trend as the applied force increases from 19 N to 250 N. At lower forces (19 N to 96 N), both materials exhibited relatively low mean strain values with no significant difference between them (*p* > 0.05). However, from 115 N onward, polyamide RPDs consistently showed higher mean strain values compared to metal acrylic RPDs.

**Fig. 7:**
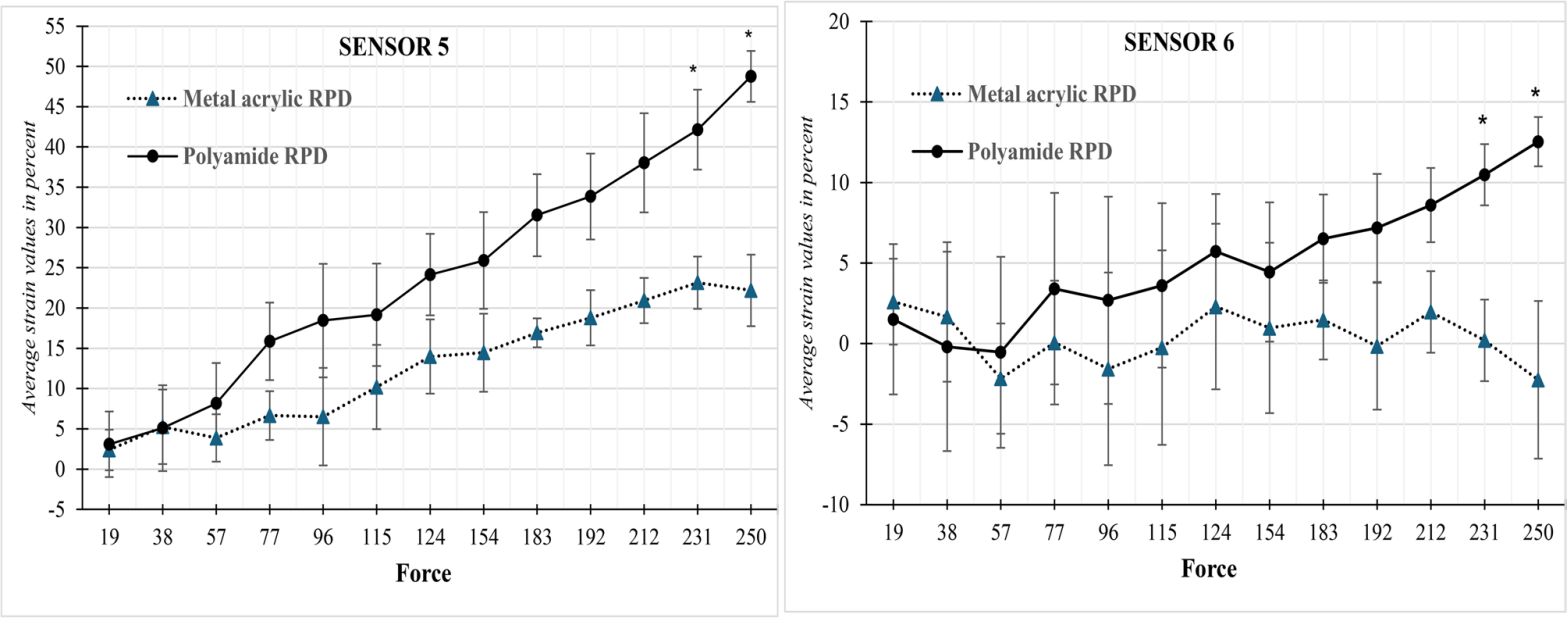
Comparison of transmitted strain between Metal acrylic and Polyamide RPD at sensors 5 and 6

Although differences became more pronounced with increasing force, statistical significance was only reached at higher load levels. Specifically, at 231 N and 250 N, the polyamide RPDs demonstrated significantly higher strain values than metal acrylic RPDs, with p-values of 0.033 and 0.008 respectively. At 212 N, the difference approached significance (*p* = 0.064), indicating a trend toward greater deformation in polyamide dentures under high stress as shown in Table 2; Fig. 7

*In Sensor 6*, the mean strain values for both metal acrylic and polyamide removable partial dentures (RPDs) were evaluated across increasing force levels, from 19 N to 250 N. Throughout the lower force range (19–183 N), both types of RPDs exhibited relatively small and statistically non-significant differences in strain (*p* > 0.05). In fact, at some points, such as 57 N and 96 N, the metal acrylic RPDs even recorded slightly negative or minimal strain values, indicating limited deformation under load.

As the applied force increased beyond 183 N, the polyamide RPDs began to show consistently higher mean strain values compared to the metal acrylic RPDs. This difference became statistically significant at 231 N (*p* = 0.031) and 250 N (*p* = 0.045), indicating that polyamide dentures deform significantly more than metal acrylic ones under high stress. For example, at 250 N, the polyamide RPDs reached a mean strain of 12.54 ± 2.65 compared to − 2.25 ± 8.47 in metal acrylic RPDs as in Table 2; Fig. 7

## Discussion

The result of this study shows stresses recorded at all sensors, like polyamide distal extension RPD, transmit higher, homogenous stresses, i.e., the force was evenly distributed on the entire ridge. The null hypothesis is rejected, indicating that polyamide RPDs transmit stress more evenly, which may reduce residual ridge resorption compared to metal-acrylic RPDs. The internal validity is strong, with appropriate use of strain gauges, standardized denture design, and controlled loading conditions ensuring accurate stress analysis [15–17]. Externally, the findings align with previous studies reporting better flexibility and stress modulation in polyamide dentures. Similar research supports that uneven stress in rigid frameworks contributes to ridge resorption, confirming the clinical relevance and generalizability of the results to real-world prosthodontic practice [18–20]. Whereas the stresses transmitted in metal acrylic distal extension, RPDs were inhomogeneous, i-e the force was unevenly distributed on the entire ridge. Higher stresses were concentrated at sensor 1, sensor 3, sensor 4, and sensor 5. Sensor 2 shows lower stress concentration, and the least stress was observed at sensor 6. This was due to the different positions of each sensor. In another study, stresses applied to edentulous ridges and in the Polyamide RPD and Cobalt Chromium RPD were compared using 3D FEM. As support of Polyamide RPD is mainly provided by the edentulous ridges due to lack of rests it seems reasonable that maximum stress from Polyamide RPD is applied on the residual ridges and less stresses were applied on the teeth. As there were rests in Cobalt chromium RPD, the maximum stress was applied on the rest seats of the teeth, and less stress was applied to edentulous ridges. So, it may be assumed that the stresses on the edentulous ridges in the Polymide RPD without any rests would be more than those in the Cobalt Chromium RPD model, which had rests. However, vice versa is true; the amount of stress on the edentulous ridges in the Polyamide RPD model was much less than that of the Cobalt Chromium RPD model. These significant differences in stresses applied on edentulous ridges may be attributed to the elasticity and stress-breaking properties of nylon-based RPDs. In other words, polyamide RPD can absorb stresses in itself and will transfer too little stress on the ridge and teeth [16, 21–24]. Figure 5

Mousa et al. [17] explained that the flexibility of the major connector in flexible RPDs plays the role of stress breaker. According to them, the flexible base of these prostheses can bend during force application. It may deform and float on the underlying tissues, leading to the release of force within the structure of the prosthesis base and, consequently its application on a large surface of an edentulous ridge. According to our study, homogenous stresses were transferred in polyamide RPDs as compared to metal acrylic RPDs in which stresses transferred were in-homogenous. Takabayashi [18] also pointed out that thermoplastic prostheses, through force distribution at a more extensive surface, can relieve the pain caused by excessive local pressure from metal acrylic RPDs onto the residual ridge. Both research by Mousa and Takabayashi were utterly consistent with the results of this study. In metal acrylic, RPD forces cannot distribute in the structure of the base because of its hardness and rigidity [19]. In fact, while applying a vertical force to tissues and without considering horizontal displacements of the RPD base, metal acrylic RPD transmits one part of the force to the abutment teeth and the other part to residual ridges because of its rigidity [20–22]. It was clearly shown in this study that the Polyamide RPD can distribute the forces in a broader surface and can transmit lower and even forces to each unit of the residual ridge surface as compared to metal acrylic RPD due to high elasticity and flexibility. Kumar et al. [21] also described that flexible (polyamide) denture provides a higher standard of function by using the material’s flexibility to balance masticatory forces over the entire supporting ridge instead of individual support points. Distribution of forces in polyamide dentures is even which results in delayed bone resorption [23, 24]. Figure 6

These results were not supported by the study of Iwata [25] and Wadachi et al. [26]; they investigated and compared the load exerted by non-metal clasp dentures and conventional PMMA dentures and found that the load exerted on the residual ridge by these non-metal clasp dentures is potentially more excessive than that with a conventional removable partial denture. Šćepanović et al. [27] compared load transfer characteristics of unilateral traditional PMMA distal extension removable partial denture with poly-acetal RPD with resin components. The highest stress was observed in thermoplastic removable partial dentures. The lowest stress was observed with a traditional metal framework when the load was applied bilaterally. The same results were observed but with higher intensity with the poly-acetal framework when unilateral load was applied [28–30]. Figure 7

Another study showed similar results in which thermoplastic dentures were compared with conventional PMMA dentures. The comparison was performed by measuring pressure (100Nload) applied under the denture base. Polyamide resin dentures showed higher subsidence exerted with the highest pressure on underlying mucosa [31]. Zarrati et al. [32] evaluated photo-elastic stress patterns produced by various designs of bilateral distal extension RPDs and reported the most favorable force distribution recorded with RPI system cast partial dentures and occlusal load concentrated stresses near the crest of the ridge. Different studies investigated the pressure transmission area and maximum transmission(load) area of thermoplastic resin denture base material under impact load. Three groups, one polycarbonate, one ethylene propylene (Duraflex), and another polyamide (valplast), were compared with traditional PMMA resin denture base material [33, 34] According to the results of the current study, PMMA showed a larger pressure transmission area and higher maximum pressure transmission area than other groups. In this study, the stresses transmitted by metal acrylic distal extension RPDs were low but uneven, due to which more ridge resorption can occur, and stresses transmitted by polyamide distal extension RPDs were higher but even which are less damaging to the bone and tissues.

## Conclusion

The investigation of stresses transmitted in polyamide RPDs were homogenous, which means there was an even distribution of forces on the ridge. Whereas stresses transmitted in metal acrylic distal extension, RPD’s were not homogenous which means there was an uneven distribution of forces on the ridge. When higher but even stresses are transmitted there is less damage to the surrounding tissues and bone. Lesser but uneven stresses aggravate residual ridge resorption. The study implications are polyamide RPDs show more even stress distribution than metal-acrylic RPDs, potentially reducing ridge resorption and improving patient comfort. This supports using polyamide as a better material choice for distal extension cases in clinical practice.

## Supplementary Information

Supplementary Material 1.

## Data Availability

The datasets used and/or analyzed during the current study are available from the corresponding author on reasonable request.
